# From Antibodies to Artificial Intelligence: A Comprehensive Review of Diagnostic Challenges in Hashimoto’s Thyroiditis

**DOI:** 10.7759/cureus.54393

**Published:** 2024-02-18

**Authors:** Nikhil Deep Kolanu, Naimel Ansar Awan, Ayesha Imran Butt, Taufiqa Reza, Mohammed Khaleel I.KH. Almadhoun, Taher Janoowala, Syed Faqeer Hussain Bokhari, Zukhruf Zain, Tanzila Sharif, Lokesh Chauhan, Jinal Choudhari

**Affiliations:** 1 Medicine, China Medical University, Shenyang, CHN; 2 Medicine, Nawaz Sharif Medical College, Gujrat, PAK; 3 Medicine, Allama Iqbal Medical College, Lahore, PAK; 4 Medicine, Avalon University School of Medicine, Youngstown, USA; 5 Medicine and Surgery, Mutah University, Karak, JOR; 6 Medicine, Saifee Hospital, Karachi, PAK; 7 Surgery, King Edward Medical University, Lahore, PAK; 8 Family Medicine, Aga Khan University Hospital, Karachi, PAK; 9 General Practice, Fatima Jinnah Medical University, Lahore, PAK; 10 Medicine, Sarojini Naidu Medical College, Agra, IND; 11 Research & Academic Affairs, Larkin Community Hospital, Miami, USA

**Keywords:** optimized diagnostic criteria, machine learning, etiology, seronegative hashimoto thyroiditis, clinical presentation, autoimmune thyroid diseases, diagnostic challenges, thyroid peroxidase antibodies, hypothyroidism, hashimoto thyroiditis

## Abstract

Hashimoto’s thyroiditis (HT) poses diagnostic challenges due to its diverse clinical presentation and the intricacies of autoimmune thyroid diseases. This comprehensive narrative review explores the evolving landscape of diagnostic challenges in HT, aiming to provide a thorough understanding of the complexities involved in its diagnosis. The diagnostic criteria for HT involve a multifaceted approach, including clinical features, laboratory findings, and imaging studies. Serum antibodies against thyroid antigens, primarily thyroperoxidase (TPO) and thyroglobulin, play a crucial role in confirming the autoimmune nature of the disease. However, seronegative HT adds complexity by presenting without detectable antibodies. The significance of addressing diagnostic challenges lies in potential delays and misdiagnoses, emphasizing the need for accurate and timely intervention. The review explores future directions, emphasizing molecular and cellular aspects, genetic factors, and the emerging field of thyroid regeneration. Standardized diagnostic criteria are essential, considering the subjective nature of the current process. The heterogeneity of disease manifestations complicates targeted treatments, necessitating a deeper understanding of clinical presentations and underlying pathophysiology. Future research directions and challenges outlined in this review contribute to advancing our understanding and improving diagnostic precision in HT.

## Introduction and background

Hashimoto’s thyroiditis (HT) is an autoimmune disorder characterized by chronic inflammation of the thyroid gland, leading to hypothyroidism [1]. The hallmark of HT is the presence of thyroid peroxidase antibodies (TPOAbs) and thyroglobulin antibodies, contributing to the destruction of thyroid tissue and subsequent hypothyroidism [2]. It is the most common cause of hypothyroidism in iodine-sufficient regions and poses diagnostic challenges due to subtle early symptoms and the gradual progression of the disease [1]. The condition, named after Dr. Hakaru Hashimoto, who first described it in 1912, primarily affects women, with an increased incidence after the age of six [1]. The prevalence rates of HT vary worldwide. A systematic review and meta-analysis found a prevalence of 7.5%, with a higher prevalence of 11.4% in low- and middle-income groups [3]. The prevalence varies with factors such as geographical location, iodine intake, and genetic predisposition. Understanding the prevalence is crucial for healthcare providers to anticipate and manage the rising cases of HT effectively.

The diagnostic challenges associated with HT stem from the diverse clinical presentation and the complexity of autoimmune thyroid diseases. While the diagnosis is commonly based on clinical symptoms of hypothyroidism and the detection of specific antibodies, the existence of seronegative HT adds a layer of complexity, with 5-10% of cases presenting without detectable antibodies [1,4,5]. This poses challenges in identifying and managing individuals with atypical or asymptomatic presentations of HT, requiring a nuanced approach in clinical practice.

The significance of exploring diagnostic challenges in HT lies in the potential delays and misdiagnoses that individuals may encounter. Early and accurate diagnosis is crucial for timely intervention and management to prevent complications associated with untreated hypothyroidism. Moreover, addressing diagnostic challenges can pave the way for improved precision in healthcare, ensuring tailored treatment strategies for diverse presentations of HT. The purpose of this comprehensive narrative review is to delve into the evolving landscape of diagnostic challenges in HT. By critically analyzing recent advancements and existing literature, the review aims to provide a thorough understanding of the complexities involved in HT diagnosis. This exploration is essential for healthcare practitioners, researchers, and policymakers to enhance diagnostic precision, ultimately improving patient outcomes and quality of life.

In the subsequent sections of this narrative review, we will further explore the etiology of HT, current diagnostic approaches, and emerging technologies that contribute to the intricate diagnostic landscape. By synthesizing evidence-based information, this review seeks to contribute to the broader understanding of HT diagnosis and inform future research directions in the field.

## Review

Etiology and clinical symptoms

HT is a chronic autoimmune condition primarily affecting the thyroid gland, leading to inflammation and eventual hypothyroidism [1,6]. The pathogenesis of HT involves dysregulation of both T-cell and B-cell immune responses [7]. Infiltration of the thyroid gland by autoreactive T lymphocytes contributes to the destruction of thyroid cells. B cells, on the other hand, are implicated in antibody production against thyroid antigens. There is a characteristic production of autoantibodies targeting thyroid-specific proteins. Anti-thyroid peroxidase (anti-TPO) and anti-thyroglobulin antibodies are commonly detected in the serum of individuals with HT, serving as diagnostic markers for the condition [8]. These antibodies contribute to the immune-mediated destruction of thyroid tissue, leading to the characteristic histological features observed in HT. The development of autoimmune thyroid diseases, including HT, is influenced by a complex interplay of genetic and environmental factors [9]. Studies suggest a strong familial association, and certain genes, including *HLA-DR* and *CTLA-4*, have been implicated in HT susceptibility [10,11]. Environmental factors also play a crucial role, with excess dietary iodine identified as a significant trigger of HT [12]. Additionally, studies highlight the impact of environmental toxicants, emphasizing the complex interplay between genetic predisposition and environmental influences in HT development [13].

HT manifests with a spectrum of clinical features, both thyroid-related and non-thyroidal. Understanding these manifestations is essential for accurate diagnosis and comprehensive patient care [1,14]. Common signs include fatigue, weight gain, sensitivity to cold, dry skin, and constipation. Additionally, individuals with HT may experience muscle weakness and joint pain, reflecting the impact of reduced thyroid hormone levels on various physiological functions. HT is often associated with various autoimmune conditions. For example, there is an increased prevalence of coexisting autoimmune disorders such as rheumatoid arthritis, type 1 diabetes, and lupus among individuals with HT [15,16]. The shared autoimmune etiology suggests a complex interplay of genetic and environmental factors influencing the immune system's response. The chronic nature of HT and its associated hypothyroidism can have significant effects on mental health [17]. Patients may experience symptoms such as depression, anxiety, and cognitive impairment. The relationship between thyroid function and mental health underscores the importance of monitoring and addressing the psychological well-being of individuals with HT.

Diagnostic criteria

In the diagnosis of HT, a multi-faceted approach involving laboratory tests and imaging studies is essential for an accurate and comprehensive assessment. Thyroid-stimulating hormone (TSH) is a crucial marker in assessing thyroid function. Elevated levels of TSH often indicate hypothyroidism, a common manifestation of HT [15]. TSH levels are routinely measured to gauge the overall thyroid function and guide further diagnostic evaluations. Measurement of free thyroxine (FT4) and free triiodothyronine (FT3) provides insights into the actual thyroid hormone levels circulating in the bloodstream. In HT, decreased levels of these hormones may be observed, reflecting the impairment of thyroid function associated with the autoimmune destruction of thyroid tissue. The presence of anti-TPO and anti-thyroglobulin (anti-TG) antibodies is indicative of autoimmune thyroiditis, confirming the immune-mediated nature of HT [1,15]. These antibodies are often elevated in individuals with HT and play a crucial role in the diagnostic criteria.

Ultrasonography is a valuable imaging modality for visualizing the thyroid gland's structure. In HT, characteristic findings include a heterogeneous echotexture and the presence of diffuse hypoechogenicity. Ultrasound aids in assessing the size, shape, and texture of the thyroid, contributing to the overall diagnostic evaluation [18,19]. In the context of HT, fine-needle aspiration biopsy (FNAB) can help confirm the autoimmune nature of the disease, especially when coupled with the presence of lymphocytic infiltrates and germinal centers in the histopathological analysis [20,21].

Challenges in diagnosis

The diagnosis of HT can be challenging, particularly in its early stages, when overt symptoms may be absent. Subclinical presentations often lack specific clinical manifestations, making it difficult to diagnose the disease solely based on symptoms [1,14]. Diagnostic reliance on laboratory tests and imaging becomes paramount in such cases, emphasizing the need for a comprehensive diagnostic approach. Given the subtle nature of early HT, periodic screening becomes imperative, especially in populations at a higher risk. Individuals with a family history of autoimmune thyroid diseases or other autoimmune conditions should undergo regular thyroid function tests and antibody assessments [22]. This proactive approach enhances the likelihood of identifying HT in its subclinical stages, facilitating timely intervention and management. Furthermore, the clinical course of HT may involve periods of thyroid gland inflammation, leading to transient hyperthyroidism known as Hashitoxicosis [23,24]. This phase may present with symptoms such as palpitations, anxiety, and weight loss, further complicating the diagnostic process. The variability in symptomatology necessitates a comprehensive understanding of the clinical spectrum of HT to enhance diagnostic accuracy.

HT shares clinical features with Graves' disease, another autoimmune thyroid disorder. Both conditions may present with symptoms of thyroid dysfunction, such as fatigue and weight changes [1]. However, distinguishing between the two is crucial for appropriate treatment planning. HT is characterized by hypothyroidism, while Graves' disease is associated with hyperthyroidism. Laboratory tests, including thyroid function panels and antibody assays, play a pivotal role in differentiation. HT can also coexist with nodular thyroid disease, further complicating the diagnostic landscape [25,26]. Nodules may be identified through imaging studies, and their presence necessitates a thorough evaluation to discern whether they are benign or harbor malignant potential. FNAB serves as a valuable tool in this scenario, aiding in the determination of nodular pathology and guiding appropriate management strategies.

Drug-induced thyroid dysfunction can manifest as hypothyroidism, mimicking HT symptoms. Certain medications, such as amiodarone, lithium, and interferons, may adversely affect thyroid function [27]. Amiodarone, an antiarrhythmic drug, contains iodine and can cause both hypothyroidism and hyperthyroidism [28]. Lithium, commonly used for psychiatric disorders, can induce hypothyroidism. Interferons, employed in various medical conditions, may also disrupt thyroid function [29]. Distinguishing between HT and drug-induced thyroid dysfunction requires a thorough patient history, including medication use, and specific thyroid function tests. The diagnosis should consider temporal associations between drug initiation and the onset of thyroid dysfunction.

Central hypothyroidism, arising from dysfunction in the pituitary or hypothalamus, adds complexity to the differential diagnosis [30]. While HT primarily results from autoimmune thyroiditis, central hypothyroidism involves inadequate stimulation of the thyroid gland due to pituitary or hypothalamic dysfunction. Clinical differentiation involves assessing the levels of TSH and free thyroid hormones (FT4 and FT3). In HT, TSH levels are typically elevated, reflecting the thyroid's struggle to produce hormones. Conversely, central hypothyroidism may present with low or normal TSH levels, indicating a dysfunction in the pituitary or hypothalamus. Additional investigations, such as imaging studies and dynamic testing, aid in confirming central hypothyroidism. This comprehensive approach ensures accurate discrimination between HT and central hypothyroidism, guiding appropriate therapeutic interventions.

Seronegative HT

In the realm of HT, a subset of cases presents a diagnostic challenge known as seronegative HT, accounting for approximately 5-10% of all HT cases [5]. Unlike typical HT cases, where the diagnosis relies on the presence of TPO antibodies, seronegative cases lack detectable levels of these antibodies, making the diagnosis more intricate [5]. Understanding the occurrence of seronegative HT is crucial for clinicians and researchers alike. This phenomenon suggests that relying solely on antibody testing might lead to the underdiagnosis of HT, particularly in cases where patients exhibit clinical symptoms of hypothyroidism but lack the characteristic antibody elevation [31]. The occurrence of seronegative HT underscores the need for a comprehensive diagnostic approach, incorporating clinical evaluation, imaging studies, and other available tests to establish an accurate diagnosis [5].

Diagnostic implications in seronegative HT extend beyond the challenge of identification. Patients with seronegative HT may present with a milder form of hypothyroidism at the time of diagnosis compared to their seropositive counterparts [32]. This highlights the importance of recognizing the distinct clinical profile associated with seronegative cases. Additionally, the diagnostic journey for seronegative HT patients may involve a more thorough exploration of clinical symptoms and reliance on alternative diagnostic modalities beyond traditional antibody testing [5]. Clinical considerations in seronegative HT extend to the management and treatment of affected individuals. Since these cases may initially present with milder hypothyroidism, monitoring for disease progression and adjusting treatment accordingly have become essential aspects of patient care [32]. Clinicians should also remain vigilant in considering the possibility of seronegative HT in patients with suggestive symptoms, even when standard antibody tests yield negative results.

Painful HT

Painful HT (pHT) represents a rare and challenging manifestation of HT, characterized by pain and tenderness in the thyroid gland. A literature review by Peng et al. explores the landscape of pHT through case reports and studies, providing insights into its rarity and the ambiguity surrounding optimal treatment strategies [33]. Notably, the review indicates that approximately 50.8% of reported cases had a known history of thyroid disease, including HT [33]. Diagnostic challenges in pHT arise from its atypical presentation, often mimicking other thyroid disorders or conditions. Case studies, such as the one reported by Seo et al., underscore the importance of considering pHT in the differential diagnosis of patients presenting with pain and tenderness in the thyroid region [34]. In this specific case, the patient's erythrocyte sedimentation rate (ESR) played a crucial role in confirming the inflammatory nature of the condition [34]. Biochemical aspects of pHT involve exploring markers that can aid in diagnosis and differentiation from other thyroid disorders. While traditional thyroid function tests may provide valuable information, additional inflammatory markers, such as ESR or C-reactive protein (CRP), may be instrumental in supporting the diagnosis, as seen in the case study by Seo et al. [34]. Understanding the inflammatory nature of pHT is pivotal for clinicians in formulating an accurate diagnosis and devising an appropriate treatment plan.

Advances in diagnostic technologies 

Recent research has delved into microRNA profiles as potential molecular markers for HT. MicroRNAs play a crucial role in gene expression regulation, and their dysregulation has been linked to various autoimmune diseases, including HT. Identifying specific microRNA signatures associated with HT could offer a non-invasive method for early diagnosis and monitoring disease progression [35]. Genetic markers have also been investigated to assess susceptibility to HT. Studies aim to identify specific genetic variations or polymorphisms that may predispose individuals to developing HT. Understanding the genetic basis of susceptibility contributes to the development of targeted diagnostic approaches, especially in populations with a familial history of autoimmune thyroid diseases [5].

Artificial intelligence facilitates the integration of clinical and imaging data, creating a comprehensive diagnostic approach. By analyzing patient-specific parameters alongside imaging features, these algorithms enhance the accuracy of HT diagnosis. For instance, computer-aided diagnostic techniques utilizing grayscale features in ultrasound imaging contribute to a more objective and reproducible identification of HT [36]. Machine learning (ML) applications have also shown promise in the field of diagnosing thyroid diseases, offering innovative approaches to enhance accuracy and efficiency in identification. Various studies explore the optimization of machine learning models to predict thyroid diseases with high accuracy [37-39]. An efficient ML approach has been designed, showcasing its potential as a valuable tool in predicting thyroid diseases with precision [40]. The use of selective features and advanced methods, such as deep learning, further contributes to the effectiveness of machine learning in thyroid disease prediction [41,42].

Despite the advancements, implementing machine learning in thyroid disease diagnosis is not without challenges. Critiques revolve around the interpretability of complex models and the need for validation in diverse patient populations. The use of machine learning raises concerns about the "black box" nature of some models, making it challenging for clinicians to understand the decision-making process. This lack of interpretability can hinder trust and acceptance in clinical settings. Moreover, the reliance on extensive datasets for training ML models introduces potential biases, as the data may not fully represent the diversity of thyroid disease presentations. Generalizing the findings to different populations or accounting for rare conditions becomes a notable challenge. Additionally, the integration of machine learning into routine clinical practice requires overcoming issues related to data privacy, ethical considerations, and standardization across healthcare systems.

Future directions and challenges

Future directions involve exploring molecular and cellular aspects to decipher underlying mechanisms. Investigating the role of genetic factors, environmental triggers, and the immune response will contribute to a more comprehensive understanding of disease etiology. The emerging field of thyroid regeneration holds promise, with ongoing studies focusing on tissue engineering and regenerative medicine [43,44]. Advancements in these areas could potentially lead to novel therapeutic strategies, aiming not only to manage symptoms but also to restore thyroid function.

Challenges include the need for standardized diagnostic criteria. The current diagnostic process, reliant on clinical features and serum antibodies, can be subjective and may not capture the full spectrum of the disease. Future efforts should aim to establish uniform and precise criteria to ensure an accurate and timely diagnosis. Additionally, the heterogeneity of the disease manifestations poses a challenge in developing targeted treatments. Tailoring therapeutic approaches based on individual patient profiles requires a deeper understanding of the diverse clinical presentations and underlying pathophysiology. Moreover, exploring the implications of HT beyond the thyroid gland is an emerging challenge. The association between HT and dyslipidemia in childhood highlights the systemic impact of the disease, necessitating a holistic approach to patient care. Future studies should delve into the extra-thyroidal effects of HT and their implications for overall health.

## Conclusions

The diagnostic challenges inherent in HT are due to its diverse clinical presentation and autoimmune complexities. This review emphasizes the importance of accurate and timely diagnosis, exploring the multifaceted diagnostic criteria involving clinical features, laboratory findings, and imaging studies. Serum antibodies, particularly TPO and thyroglobulin, play a crucial role in confirming the autoimmune nature of HT, but the existence of seronegative cases adds complexity. The article discusses challenges in diagnosis, including subclinical presentations, differentiation from other thyroid disorders, and drug-induced thyroid dysfunction. It delves into emerging technologies such as microRNA profiling, genetic markers, and artificial intelligence, offering promising avenues for improved diagnostic precision. Future directions in HT research, such as molecular exploration and the potential of thyroid regeneration, are outlined, along with the persistent challenges of standardized diagnostic criteria and the need for a holistic approach to patient care, considering extra-thyroidal effects. Overall, this review contributes valuable insights for healthcare practitioners, researchers, and policymakers, aiming to advance our understanding of HT and enhance diagnostic accuracy.
